# The role of leisure-time physical activity in maintaining cervical lordosis after anterior cervical fusion and its impact on the motor function in patients with hirayama disease: a retrospective cohort analysis

**DOI:** 10.1186/s12891-023-07038-w

**Published:** 2023-11-21

**Authors:** Kaiwen Chen, Yang Yang, Xiaoqin Wang, Yu Zhu, Feizhou Lyu, Jianyuan Jiang, Xinlei Xia, Chaojun Zheng

**Affiliations:** 1 Department of Orthopedics, Huashan Hospital, Fudan University, 12 Mid- Wulumuqi Road, Shanghai, 200040 China; 2 Department of Radiology, Huashan Hospital, Fudan University, Shanghai, 200040 China; 3 Department of Nursing, Huashan Hospital, Fudan University, Shanghai, 200040 China; 4 Department of Physical Medicine and Rehabilitation, Upstate Medical University, State University of New York at Syracuse, Syracuse, NY 10212 USA; 5 Department of Orthopedics, The Fifth People’s Hospital, Fudan University, Shanghai, 200240 China

**Keywords:** Hirayama disease, Spinal curvatures, Physical activity, Motor unit, Surgery

## Abstract

**Background:**

Surgical treatment has been increasingly performed in Hirayama disease (HD) patients to limit excessive neck flexion and restore cervical lordosis. However, postoperative recurrence of cervical lordosis loss may restart the progress of HD. Many studies have demonstrated a relationship between neck muscle strength and cervical lordosis, and it is widely accepted that leisure-time physical activity (LTPA) can increase muscle strength. However, there are few reports about the correlation between LTPA and maintenance of postoperative cervical curvature.

**Objective:**

To quantify the cervical lordosis and motor function before and after operation in HD patients and to analyze the impact of postoperative LTPA levels on the changes in these measurements.

**Methods:**

C2-7 Cobb were measured in 91 HD patients before, 2–5 days and approximately 2 years after operation. Motor unit number estimation (MUNE) and handgrip strength (HGS) were performed in all patients before and approximately 2 years after operation, and both cross-sectional area and fatty infiltration of posterior cervical muscles were measured in 62 patients. Long-form international Physical Activity Questionnaire and its different domains was administered to all patients at postoperative 2-year assessments.

**Results:**

The C2-7 Cobb was larger immediately and approximately 2 years after operation than that at preoperative assessment (P < 0.05). The preoperative to postoperative change in C2-7 Cobb was associated with postoperative changes in the symptomatic-side HGS and bilateral MUNE measurements (P < 0.05). Importantly, the patients performing LTPA had greater improvements in C2-7 Cobb from immediate to approximately 2 years after operation and greater C2-7 Cobb at last follow-up than those without LTPA, and postoperative improvements in both symptomatic-side MUNE measurements and symptomatic-side HGS were also greater in the former than in the latter (P < 0.05).

**Conclusions:**

Postoperative LTPA has a positive effect on recovery/maintenance of cervical lordosis after operation, which may alleviate the motor unit loss of distal upper limbs in HD patients. Therefore, postoperative LTPA may be beneficial for postoperative rehabilitation or early conservative treatment of HD patients.

**Supplementary Information:**

The online version contains supplementary material available at 10.1186/s12891-023-07038-w.

## Introduction

Hirayama disease (HD) is a juvenile-onset and male-prone disorder that is characterized by unilateral or asymmetric weakness and amyotrophy of the hand and forearm muscles without sensory deficits [1].

**Fig. 1:**
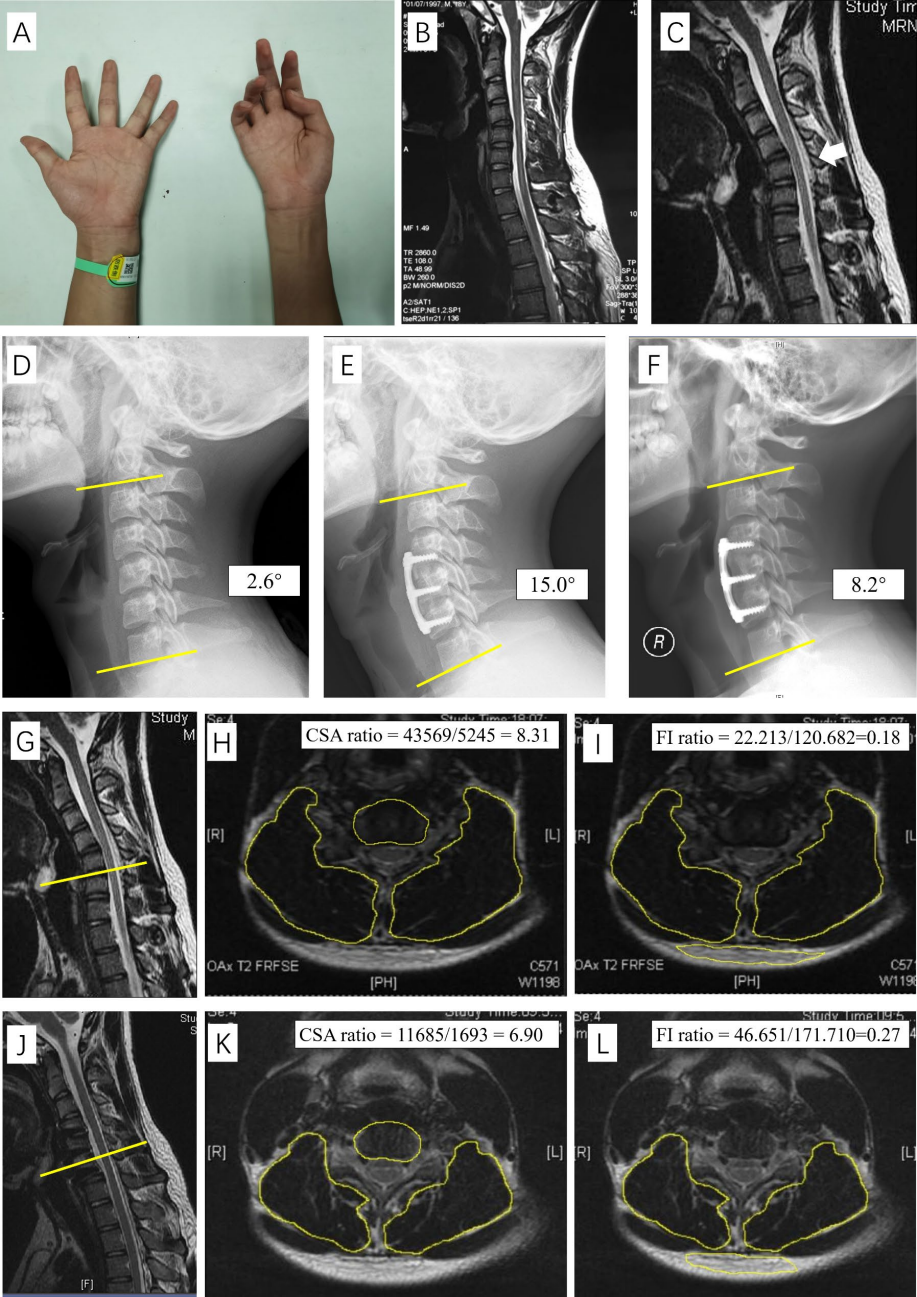
Preoperative and postoperative imaging assessments in a patient with known Hirayama disease. **A**: Significant amyotrophy of the intrinsic hand on the right side. **B**: In the neck-neutral position, no significant abnormality on cervical MRI is observed. **C**: In the neck-flexion position, forwards-shifting of the posterior dura and a crescent-shaped LOA behind the posterior dura on MRI are shown (white arrow). **D**: Preoperative assessments of cervical sagittal alignment (C2-7 Cobb method). **E**: Postoperative immediate assessments of cervical sagittal alignment (C2-7 Cobb method). **F**: Postoperative 2-year assessments of cervical sagittal alignment (C2-7 Cobb method). **G**: Preoperative localization of the C5-6 segment on neck-flexion MRI. **H**: Preoperative measurements of the cross-sectional area of the posterior cervical muscles. **I**: Preoperative measurements of the fatty infiltration of the posterior cervical muscles. **J**: Postoperative localization of the C5-6 segment on neck-flexion MRI. **K**: Postoperative measurements of the cross-sectional area of the posterior cervical muscles. **L**: Postoperative measurements of the fatty infiltration of the posterior cervical muscles. **MRI**: Magnetic resonance imaging. **LOA**: Loss of dorsal dural attachment from the pedicle

The current hypothesis for the etiology of HD mainly implicates chronic ischemia of cervical motor neurons/ventral roots caused by neck flexion [1, 2], and this condition is caused by abnormal forward-shifting of the lower cervical posterior dura on neck flexion possibly due to loss of dorsal dural attachment from the pedicle (LOA) (Fig. 1). Importantly, both Chen et al. and Zheng et al. have demonstrated this characteristic LOA can also occur in the neck-neutral magnetic resonance imaging (MRI) in HD patients; thus, loss of cervical lordosis may also be involved in the pathogenic process of HD [3, 4]. Based on these hypotheses, surgical treatment (e.g., anterior cervical fusion, ACF) has been increasingly performed in HD patients since ACF procedures can not only permanently limit excessive neck flexion but also restore cervical lordosis [1, 5]. However, Xu et al. observed that there was an abnormal increase in the range of motion of cervical segments in HD patients [6], and a recently published finite element analysis further demonstrated that cervical fusion will increase this pre-existing hypermobility of surgical adjacent segments in HD patients, consequently stimulating adjacent segment degeneration [7], which may cause the recurrence of cervical lordosis loss soon after operation. Importantly, postoperative loss of cervical sagittal alignment may not only reversely accelerate adjacent segment degeneration [8, 9] but also restart the progress of HD because of the reappearance of pathogenic factors. Therefore, it is of clinical significance to long-term maintain physiological lordosis after operation in HD patients.

Accumulating evidence has demonstrated that there is a close relationship between posterior neck muscle strength and cervical physiological lordosis [10–12], and many previous studies have demonstrated that increasing leisure-time physical activity (LTPA) can increase trunk muscle strength. According to the previous studies [13, 14], LTPA can be defined as bodily movement that results in energy expenditure mainly from sports, conditioning, exercise and their related activities. However, there have been few reports about the correlation between LTPA levels and maintenance of cervical physiological curvature after operation, especially in patients with HD.

Hence, the aim of this study was to quantify the cervical lordosis and motor function before and after operation in HD patients and to analyze the impact of postoperative LTPA levels on the changes in these measurements.

## Methods

### Participants

All HD patients were recruited at the author’s institution between July 2017 and November 2020. Informed consent was obtained from the subjects or their parents/legal guardians (for patients under the age of 18) in this study.

The inclusion criteria for HD were as follows [4, 5]: (1) insidious disease onset before 25 years of age; (2) unilateral or asymmetric weakness and amyotrophy of the upper limbs without sensory dysfunction or lower limb involvement; (3) cervical-flexion magnetic resonance imaging (MRI) demonstrating lower cervical compression resulting from forward-shifting of the posterior dura and a crescent abnormality posterior to the dura; and (4) subjects undergoing ACF procedures.

The exclusion criteria were as follows [4, 5]: previous spinal/peripheral nerve surgeries, syringomyelia, peripheral vascular disease, muscle disorders, cervical spondylotic amyotrophy, motor neuron disease, primary or concomitant disorders of neuromuscular junctions, diseases of the central nervous system, spinal tumor/deformity/trauma/infection, the conditions that would disturb the subject’s cooperation, subjects with follow up time less than 18 months and who were lost to follow-up or unwilling to follow-up.

### Sample size

Between July 2017 and November 2020, a total of 178 patients were diagnosed as HD at the author’s institution, and 24 of these 178 only accepted the conservative treatments. Furthermore, twelve HD patients underwent the previous peripheral nerve surgeries (e.g., ulnar nerve relaxation), and the other 27 HD patients were lost to follow-up or unwilling to follow-up after operation. In addition, six patients were unable to cooperate with assessments due to adverse psychological conditions (e.g., depression or anxiety). Moreover, the follow-up time in 18 HD patients was less than 18 months. Therefore, this study included 91 patients with HD.

### Setting

All methods used in this study were carried out in accordance with ‘Declaration of Helsinki’, and the study protocol was approved by Human Ethics Committees (Huashan Hospital, Fudan University, China; KY2014-268).

All HD patients in this study underwent cervical X-ray before, immediately (2–5 days) after and approximately 2 years (24.2 ± 6.0 months) after operation. Cervical MRI was performed in all patients before operation, and 55 of these 91 HD patients was re-evaluated by cervical MRI approximately 2 years after operation. The cervical MRI measurements were recorded from these 55 HD patients.

Furthermore, all HD patients underwent a handgrip strength (HGS) test (Jamar hydraulic hand dynamometer, Sammons Preston Rolyan, IL, USA) and disabilities of the arm, shoulder and hand (DASH) assessment, as well as multipoint incremental stimulation motor unit number estimation (MUNE) (Nihon Kohden MEB-9400 EMG unit, Tokyo, Japan), before and approximately 2 years after operation.

In addition, the long-form International Physical Activity Questionnaire (IPAQ) was administered to all HD patients approximately 2 years after operation, and both total and each part (work, transport, domestic and garden, and leisure physical activity) of long-form IPAQ were measured. For LTPA, HD patients reported the main type of exercises.

### Variables

#### Imaging assessments

On cervical lateral X-ray, cervical sagittal alignments in all HD patients were measured at the C2-7 level by the Cobb method. In the Cobb method, the angle between the inferior margin of C2 and the inferior margin of C7 is measured (Fig. 1).

In the 55 patients who underwent both preoperative and postoperative cervical MRI, the cross-sectional area and fatty infiltration of the posterior cervical muscles were measured on both sides of each individual based on the axial T2-weighted neck-flexion MRI (Image-J; National Institutes of Health, Bethesda, MD, USA) [15], and the mean values were recorded.

We measured the cross-sectional area of the posterior cervical muscles at the C5/6 segment (the operation of all patients included this segment), and the cervical muscularity was evaluated using the muscle-vertebral body cross-sectional area ratio multiplied by 100 (the cross-sectional area of the muscles was divided by the cross-sectional area of the vertebral body at the same level) to decrease bias (Fig. 1) [15].

For fatty infiltration, we measured the average signal intensity of the posterior cervical muscles at the C5/6 segment, and the average signal intensity of subcutaneous fat was also measured at the same segment (Fig. 1). Then, the degree of fatty infiltration at the C5/6 segment was measured by multiplying the posterior cervical muscle-subcutaneous fat ratio by 100 (the posterior cervical muscle-subcutaneous fat ratio was defined as the mean signal intensity of the muscle divided by the mean signal intensity of the subcutaneous fat) [15].

### Motor function assessments

For HGS, all patients performed three maximum attempts for each HGS measurement, with one-minute intervals of rest between each attempt. The mean value of three trials was recorded as the HGS measurement.

For MUNE (skin temperatures above 32 °C), the maximal amplitude of the compound muscle action potential (CMAP) was recorded from bilateral abductor pollicis brevis (APB), and the single motor unit potential (SMUP) was recorded from three different stimulus sites (the wrist, 4 cm proximal to the wrist and the elbow). At each stimulus site, the intensity of stimulation increased slowly until the initial, second, and third SMUPs were recorded in an all-or-nothing manner. At all three stimulation points, 9 distinct SMUPs were obtained. The following parameters were measured: maximum CMAP amplitudes, average SMUP amplitudes and number of motor units (the maximum CMAP amplitude divided by average SMUP amplitude) [16].

### Physical activity assessments

The IPAQ, a well-established self-report measure of lifestyle PA, was administered throughout the entire week prior to survey. In each part of long-form IPAQ, data were collected from specific settings across different generic items, including vigorous-intensity activities and moderate-intensity activities, walking and cycling, and were reported in metabolic equivalents of task (MET) minutes per week [13]. MET is a physiological measure expressing the energy cost of PA and is defined as the ratio of metabolic rate (and therefore the rate of energy consumption) during a specific PA to a reference metabolic rate [13]. The selected MET values were derived from work undertaken during the IPAQ reliability study undertaken in 2000–2001, and both MET values and formula for measuring IPAQ scores were presented in the supplementary Table 1.

### Statistical methods

All data were analyzed using SPSS version 27.0 (IBM, USA). The Kolmogorov-Smirnov test was used to test normally distributed data. The measurements between the different HD patient groups were compared by an independent t test or Mann-Whitney test. The measurements of HD patients before and after operation or between two different postoperative assessments were compared by paired t-tests or Wilcoxon signed ranks tests. Furthermore, the frequencies between the different HD patient groups were evaluated by the chi-squared test or Fisher’s exact test. Pearson correlation tests were used to analyze the correlation between the preoperative C2-7 Cobb and preoperative measurements of both motor function and cervical MRI assessments. The same statistical methods were also used to analyze the correlation between the IPAQ measurements and the postoperative measurements of both imaging and motor function assessments. In all instances, a P-value < 0.05 was considered statistically significant.

## Results

### Preoperative assessments

In the present study, there was a negative correlation between the C2-7 Cobb and bilateral average SMUP amplitudes (Supplementary Fig. 1). The C2-7 Cobb was also negatively associated with fatty infiltration of the posterior cervical muscles (Supplementary Fig. 2).

In the present study, fifteen (15/91, 16.5%) HD patients had LOA on preoperative neck-neutral MRI, and these patients exhibited obviously lower C2-7 Cobb than those without neck-neutral LOA (76/91, 83.5%) (Supplementary Table 2). Furthermore, the former showed higher average SMUP amplitudes and lower MUNE values on both sides, as well as lower less-symptomatic-side CMAP amplitudes, compared to the latter (Supplementary Table 2).

### Postoperative assessments

**Fig. 2:**
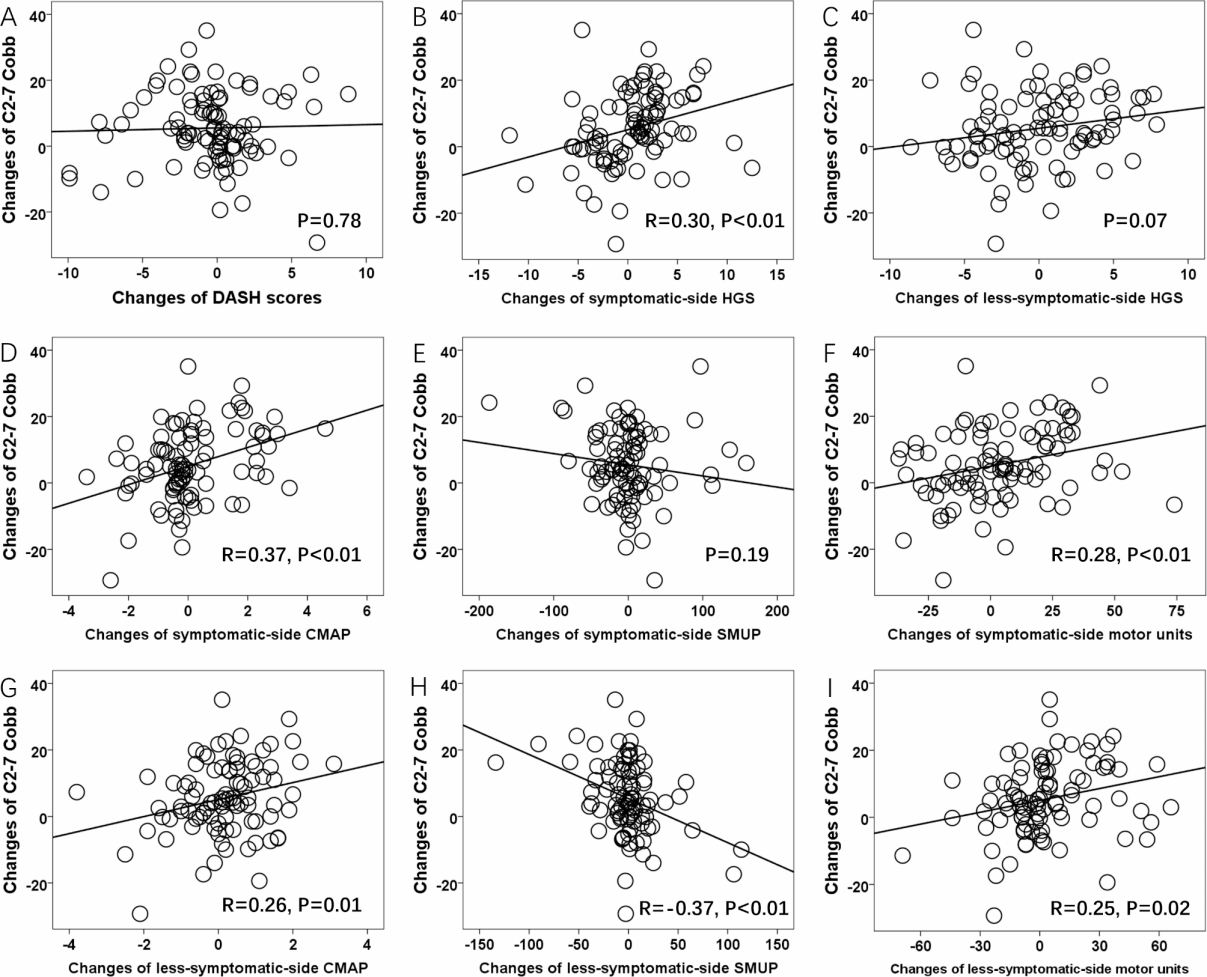
Correlation between the postoperative 2-year changes in C2-7 Cobb and the postoperative 2-year changes in the measurements of the motor functional assessments. Postoperative 2-year changes in C2-7 Cobb were positively associated with postoperative 2-year changes in symptomatic-side HGS (**B**), bilateral CMAP amplitudes (**D, G**), bilateral number of motor units (**F, I**) and less-symptomatic-side SMUP amplitudes (**H**) (P < 0.05). There was no association between the postoperative 2-year changes in C2-7 Cobb and postoperative 2-year changes in DASH scores (**A**), less-symptomatic-side HGS (**C**) and symptomatic-side SMUP amplitudes (**E**) (P > 0.05). **CMAP**: Compound muscle action potential; **SMUP**: Single motor unit potential; **HGS**: Handgrip strength; **DASH**: The disabilities of the arm, shoulder and hand outcome measure; **P**: P-values

The C2-7 Cobb was larger at both the postoperative immediate and 2-year follow-up than that in the preoperative assessments, and similar C2-7 Cobb was observed between postoperative two assessments (Table 2). Furthermore, the measurements of MUNE, HGS, DASH and cervical MRI were similar between the preoperative assessment and last postoperative follow-up (Table 2). Importantly, the pre- to postoperative 2-year change in C2-7 Cobb was associated with the changes in symptomatic-side HGS, bilateral CMAP amplitudes, bilateral MUNE values and less-symptomatic-side SMUP amplitudes within the same period (Fig. 2).

**Table 1:** The demographic and clinical characteristics of HD patients

	The patients with HD
**Number of patients**	91
**Age range (years)**	18.5 ± 3.4
**Duration (months)**	28.7 ± 32.0
**Male:Female**	85:6
**Symptomatic side (right:left)**	51:40
**Symptoms and signs (n/total patient (%))**	
Wasting	91/91 (100%)
Muscle weakness	91/91 (100%)
Fasciculation	12/91 (13.2%)
Cold paralysis	81/91 (89.0%)
Deep hyperreflexia	43/91 (47.3%)
Positive pyramidal sign	29/91 (31.9%)
**The presentation of MRI (n/total patient (%))**	
Localized cord atrophy	42/91 (46.2%)
Intramedullary high-signal lesion	15/91 (16.5%)
‘‘Snake-eye’’ appearance	9/91 (9.9%)
Neck-neutral LOA	15/91 (16.5%)
**Cervical surgical segments (n/total patient (%))**	
Number of surgical segments	2.1 ± 0.3
C4-5 and C5-6	51/91 (56.0%)
C5-6 and C6-7	32/91 (35.2%)
C3-4, C3-5 and C5-6	2/91 (2.2%)
C4-5, C5-6 and C6-7	6/91 (6.6%)

Measurements are expressed as the mean ± SD

**HD**: Hirayama disease; **LOA**: Loss of dorsal dural attachment from the pedicle; **MRI**: Magnetic resonance imaging

**Table 2:** Preoperative and Postoperative last follow-up assessments in patients with HD

	The patients with HD							
Number of patients	91							
Duration between operation and last follow-up (months)	24.2 ± 6.0 (range: 18–45)							
	Preoperative assessments		Postoperative assessments		P-values/d-values*		Differences between pre- and postoperative last follow-up	
	S side	Less-S side	S side	Less-S side	S side	Less-S side	S side	Less-S side
**MUNE assessments**								
CMAP (mV)	6.3 ± 2.8	8.4 ± 2.8	6.4 ± 2.8	8.6 ± 2.9	0.55/1.4	0.16/1.1	0.1 ± 1.4	0.2 ± 1.1
SMUP (µV)	110.5 ± 64.0	86.4 ± 44.5	110.3 ± 76.1	85.5 ± 43.5	0.96/45.8	0.78/30.8	-0.2 ± 45.8	-0.9 ± 30.8
Number of motor units	78.3 ± 57.8	130.5 ± 84.2	81.5 ± 57.7	134.2 ± 85.9	0.17/21.8	0.14/23.4	3.8 ± 21.9	3.7 ± 23.4
**Clinical functional assessments**								
HGS (Kg)	24.3 ± 11.0	32.4 ± 9.0	24.8 ± 10.1	32.4 ± 8.9	0.28/4.0	1.00/3.7	0.5 ± 4.0	0.0 ± 3.7
DASH	8.5 ± 7.0		8.1 ± 6.8		0.22/3.3		-0.4 ± 3.3	
**Imaging assessments**								
C2-7 Cobb (degrees)	6.2 ± 11.7*		12.1 ± 10.2 [11.7 ± 10.6]		< 0.01/7.2 [< 0.01/11.0]		5.5 ± 11.0	
CSA#	9.0 ± 1.9		8.7 ± 2.8		0.47/2.7		-0.3 ± 2.7	
FI#	0.23 ± 0.08		0.24 ± 0.06		0.21/0.1		0.02 ± 0.09	

Measurements are expressed as the mean ± SD

*: P-values and d-values (effect size) between the preoperative and postoperative assessments

#: These measurements of the imaging assessments were recorded from 55 of 91 HD patients.

a [b]: C2-7 Cobb at the postoperative immediate assessments [C2-7 Cobb at the postoperative 2-year assessments]

**HD**: Hirayama disease; **MUNE**: Motor unit number estimation; **CMAP**: Compound muscle action potential; **SMUP**: Single motor unit potential; **HGS**: Handgrip strength; **DASH**: The disabilities of the arm, shoulder and hand outcome measure; **CSA**: Cross-sectional area of posterior cervical muscles; **FI**: Fatty infiltration of posterior cervical muscles

Both the total and each part of long-form IPAQ assessments at postoperative 2-year assessments in patients with HD were listed in Table 3. In this study, twenty-eight (28/91, 30.8%) HD patients participating in the sports, conditioning or exercise were categorized into the LTPA group, and the main types of LTPA were listed in the Supplementary Table 3. The other sixty-three (63/91, 69.2%) HD patients without any sports, conditioning or exercise after operation were categorized into the non-LTPA group.

**Table 3:** Both total and each part of long-form IPAQ at postoperative 2-year assessments in patients with HD

	The patients with HD
**Number of patients**	91
Total IPAQ values (MET·min/w)	1703.7 ± 1086.7
Work IPAQ values (MET·min/w)	797.5 ± 1066.8
Transportation IPAQ values (MET·min/w)	433.2 ± 354.7
Domestic/Garden IPAQ values (MET·min/w)	130.8 ± 158.8
Leisure-Time IPAQ values (MET·min/w)	342.3 ± 630.6

Measurements are expressed as the mean ± SD

**HD**: Hirayama disease; **IPAQ**: International Physical Activity Questionnaire

**Fig. 3:**
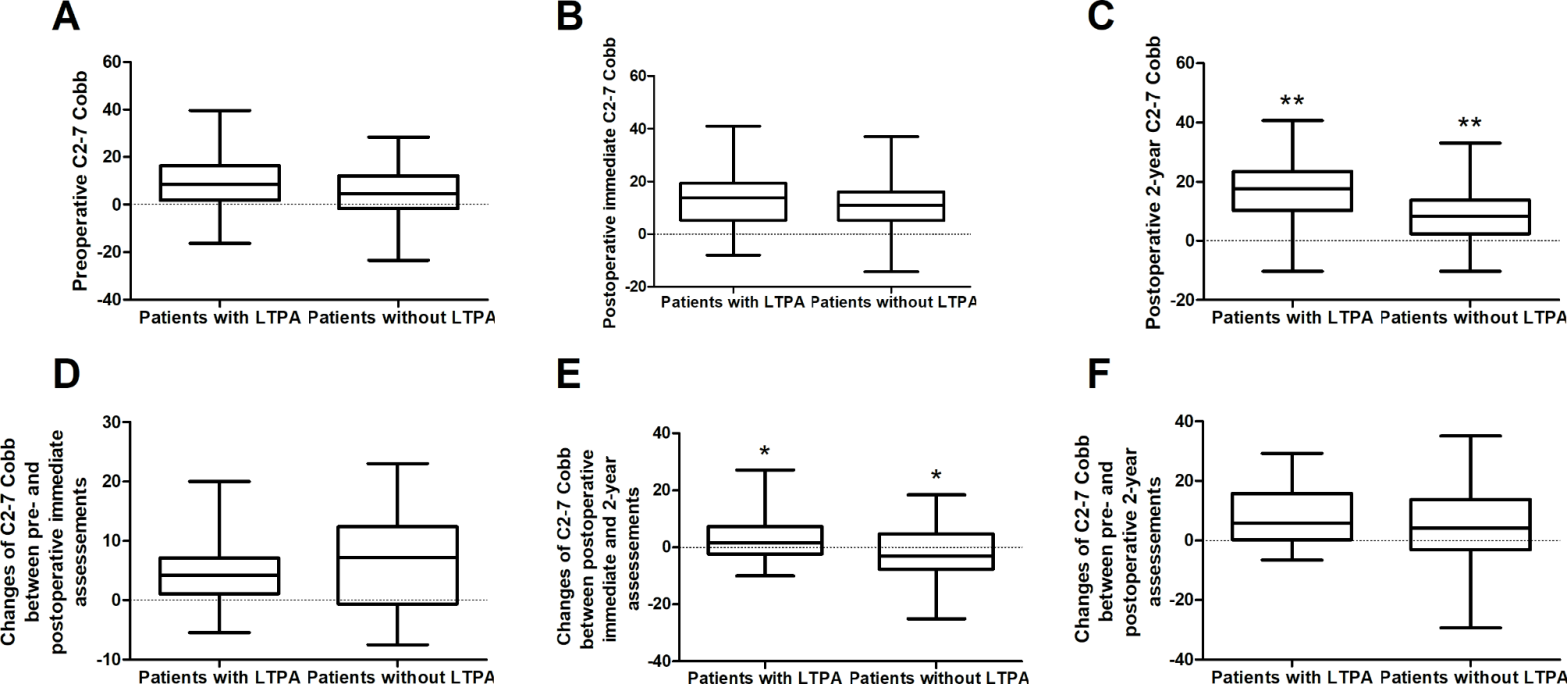
Comparison of both the C2-7 Cobbs and the changes in C2-7 Cobbs between the patients with and without LTPA (Whiskers: Min to Max). The patients without LTPA showed lower C2-7 Cobb at the postoperative 2-year follow-up compared to those with LTPA (**C**), and the HD patients performing LTPA also showed relatively greater improvements of C2-7 Cobb between postoperative immediate and 2-year assessments compared to those without LTPA (**E**). There was no difference of preoperative C2-7 Cobb (**A**), postoperative immediate C2-7 Cobb (**B**), changes of C2-7 Cobb between preoperative and postoperative immediate assessments (**D**), and changes of C2-7 Cobb between preoperative and postoperative 2-year assessments (**F**) between the patients with and without LTPA (P > 0.05). *: Significant differences between the patients with and without LTPA, *P < 0.05, and **P < 0.01. **HD**: Hirayama disease; **LTPA**: Leisure-time physical activities; **P**: P-values

**Fig. 4:**
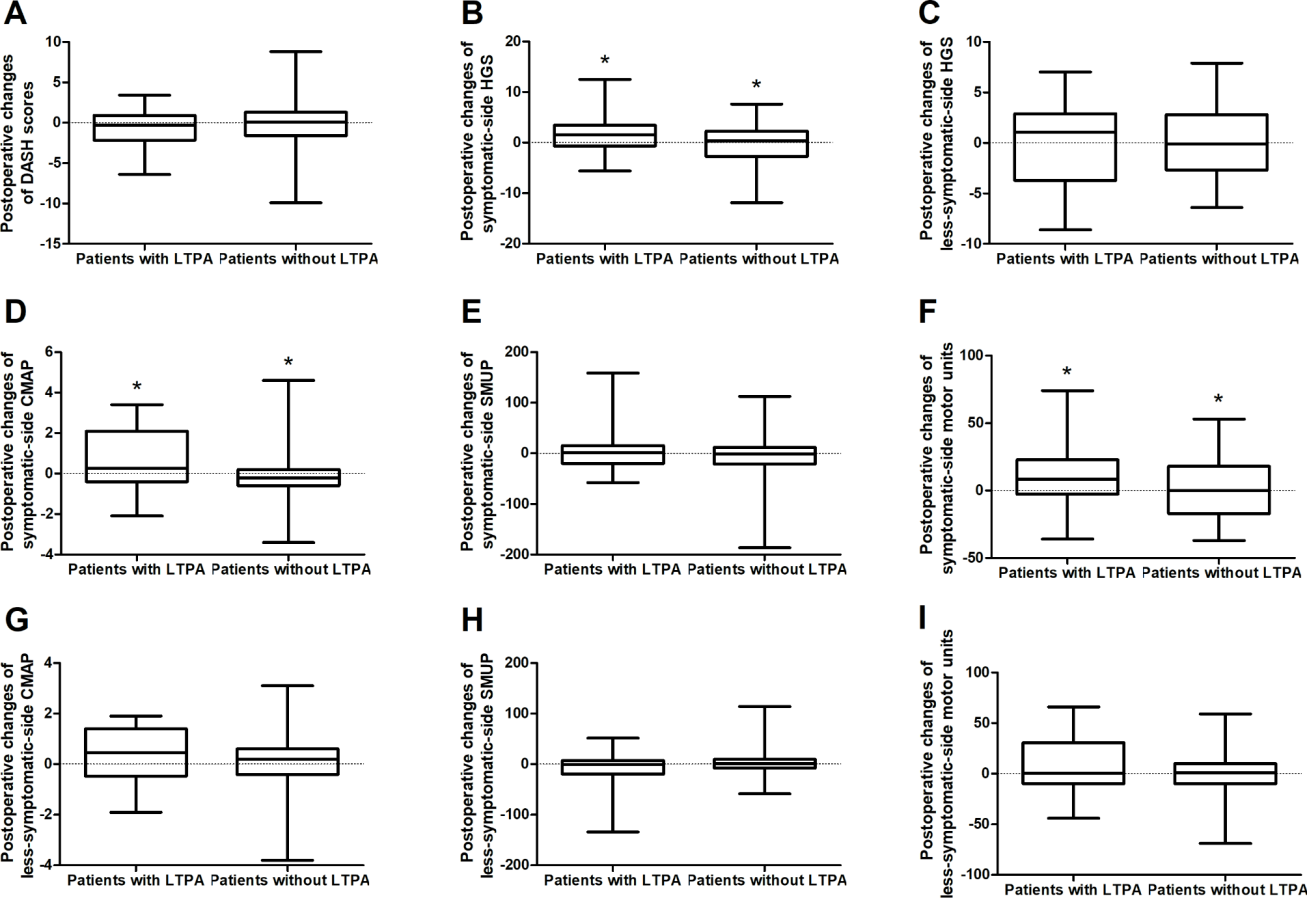
Comparison of the postoperative changes in the measurements of the motor function assessments between the HD patients with and without LTPA (Whiskers: Min to Max). The postoperative improvements in HGS (**B**), CMAP amplitudes (**D**) and the number of motor units (**F**) on the symptomatic side were greater in the patients performing LTPA than in those without LTPA. There was no difference of postoperative changes in DASH scores (**A**), less-symptomatic-side HGS (**C**), bilateral SMUP amplitudes (**E, H**), less-symptomatic-side CMAP amplitudes (**G**) and less-symptomatic-side number of motor units (**I**) between the patients with and without LTPA (P > 0.05). *: Significant differences between the patients with and without LTPA, *P < 0.05. **HD**: Hirayama disease; **LTPA**: Leisure-time physical activities. **CMAP**: Compound muscle action potential; **SMUP**: Single motor unit potential; **HGS**: Handgrip strength; **DASH**: The disabilities of the arm, shoulder and hand outcome measure; **P**: P-values

Compared with the LTPA group, non-LTPA group patients showed smaller C2-7 Cobb at the postoperative 2-year assessments (Supplementary Tables 4, Fig. 3). Importantly, compared with the latter, the former showed relatively greater improvements in C2-7 Cobb between the two postoperative assessments (Fig. 3). Furthermore, improvements in HGS, CMAP amplitudes and MUNE values on the symptomatic side were also greater in the patients having LTPA than in those not having LTPA (Fig. 4). In the LTPA group, there was no relationship between the IPAQ values for LTPA and both the postoperative measurements and changes in these measurements (Supplementary Table 5).

## Discussion

Although the preoperative measurements of both HGS and DASH were similar between the HD patients with and without neck-neutral LOA in this study, markedly different symptomatic-side MUNE measurements were observed preoperatively between these two patient groups. According to previous studies [17, 18], both muscle strength and motor activities can usually be preserved by reinnervation until 50% or more of motor units are lost. In contrast, MUNE value is independent of the motor compensatory mechanism caused by collateral reinnervation during disease advances [19]. Furthermore, recently published studies have demonstrated the relationship between the loss of cervical sagittal alignment and neck-neutral LOA in HD patients [3, 4]. Therefore, different preoperative MUNE measurements between the HD patients with and without neck-neutral LOA may suggest that loss of cervical lordosis may worsen the motor dysfunction in HD patients, which is further supported by a negative correlation between the C2-7 Cobb and average SMUP amplitudes in this study.

Similar to the previous study [5], all measurements of motor function assessments in this study were similar before and 2 years after operation, suggesting the effectiveness of ACF procedures in preventing HD progression. One of the possible reasons is that surgical treatment may limit the excessive forwards displacement of the posterior dura. According to previous studies [20, 21], imbalanced growth in HD will cause disproportional length between the spinal canal and its content, which may result in an abnormal “tight dural sac” in the neck neutral position. This abnormal “tight dural sac” is unable to stretch to fully compensate for increased cervical canal length during neck flexion, thereby resulting in abnormal forwards-shifting of lower cervical posterior dura, with consequent compressive ischemic necrosis of cervical anterior horn cells [22]. Therefore, ACF procedures may permanently eliminate this microcirculatory disturbance during neck flexion and thus prevent the progression of HD. Furthermore, loss of cervical lordosis will cause the local cervical spine to show neck-flexion-like changes in the neck neutral position, and many previous studies have demonstrated the role of loss of cervical lordosis in the pathogenesis of HD [3, 4]. Therefore, restoring cervical lordosis may be another reason for the effectiveness of ACF procedures in the treatment of HD. The positive correlation between the postoperative changes in cervical curvature and the postoperative changes in the measurements of motor function assessments in HD patients further supports this view.

According to Fig. 3, there was no difference in either the preoperative or postoperative immediate C2-7 Cobb between the patients with and without LTPA. In contrast, the former showed a larger C2-7 Cobb at the last postoperative follow-up than the latter, indicating that postoperative LTPA may encourage recovery or maintenance of cervical lordosis after operation. Significantly different recoveries of C2-7 Cobb between postoperative immediate and 2-year assessments between these two patient groups further provides evidence for this view. Importantly, changes in C2-7 Cobb between two postoperative assessments in patients without LTPA suggest that a lack of postoperative LTPA may lead to progressive loss of cervical curvature after ACF procedures.

The results of this study should be interpreted with caution since the influence of postoperative LTPA on cervical curvature is relatively small compared to that of surgical treatment. However, the results of this study demonstrated the role of LTPA in maintaining/improving cervical curvature in the medium- and long-term after operation, which may be important for HD patients undergoing ACF procedures to avoid the occurrence of adjacent segment diseases and the recurrence of HD. Furthermore, different results of postoperative improvements in MUNE measurements between the HD patients with and without postoperative LTPA may suggest that LTPA may mildly affect the postoperative motor functional recovery of HD patients. Although clinical functional improvement is very important for HD patients, mildly improved MUNE measurements may provide additional unique information for monitoring treatment outcomes and selecting treatment modalities. Another potential limitation is that it is difficult to determine which exercise is the best for the recovery/maintenance of postoperative cervical lordosis in this study since the sample size of each exercise subgroup is too small. Relatively short follow-up time may be one of the limitations of this study. However, the clinical application time of surgical treatment in Hirayama disease is not long, and the results of this study have demonstrated that the cervical curvature has been significantly different between the patients with and without postoperative exercises during the follow-up of average 2 years after operation. Furthermore, this is a retrospective study that lacks some information for HD patients (e.g., preoperative physical activity levels and spino-pelvic alignment). It is imperative to acknowledge that spino-pelvic alignment may exert a significant influence on cervical spinal alignment, and there is possible association between the preoperative physical activity habits and postoperative IPAQ results. Thus, more significant results might be obtained in a future prospective (preferably blinded) study with both more measurements (e.g., preoperative IPAQ scores and spino-pelvic alignment) and a larger sample of cases with long-term follow-up.

## Conclusion

There was a correlation between the loss of cervical sagittal alignment and motor impairments in HD patients. Importantly, postoperative LTPA was demonstrated to have positive effects on further recovery or long-term maintenance of cervical lordosis after operation, which may alleviate postoperative motor unit loss of distal upper limbs in HD patients. Therefore, postoperative LTPA may be beneficial for the postoperative rehabilitation or even early conservative treatment of HD patients.

### Electronic supplementary material

Below is the link to the electronic supplementary material.

Supplementary Material 1: Supplementary Table 1

Supplementary Material 2: Supplementary Table 2

Supplementary Material 3: Supplementary Table 3

Supplementary Material 4: Supplementary Table 4

Supplementary Material 5: Supplementary Table 5

Supplementary Material 6: Supplementary Figure 1

Supplementary Material 7: Supplementary Figure 2

Supplementary Material 8: Raw data

Supplementary Material 9: Supplementary figures legend

## Data Availability

The datasets have been presented in the article/supplementary material, further inquiries can be directed to the corresponding author/s.

## List of abbreviations

HD: Hirayama disease
LTPA: Leisure-time physical activity
MUNE: Motor unit number estimation
HGS: Handgrip strength
LOA: Loss of attachment
MRI: magnetic resonance imaging
ACF: anterior cervical fusion
DASH: disabilities of the arm, shoulder and hand
CMAP: Compound muscle action potential
APB: Abductor pollicis brevis
SMUP: Single motor unit potential
IPAQ: International Physical Activity Questionnaire

## Declarations

All authors declare that the study protocol was approved by Human Ethics Committees (Huashan Hospital, Fudan University, China; KY2014-268). Informed consent was obtained from the subjects or their parents/legal guardians (for patients under the age of 18) in this study. There was no illiterate participant who was involved in this study.

## References

[1] Lyu F, Zheng C, Wang H (2020). Establishment of a clinician-led guideline on the diagnosis and treatment of Hirayama Disease using a modified Delphi technique. Clin Neurophysiol.

[2] Hirayama K, Tomonaga M, Kitano K (1987). Focal cervical poliopathy causing juvenile muscular atrophy of distal upper extremity: a pathological study. J Neurol Neurosurg Psychiatry.

[3] Chen CJ, Hsu HL, Tseng YC (2004). Hirayama flexion myelopathy: neutral-position MR imaging findings–importance of loss of attachment. Radiology.

[4] Chen K, Yang Y, Sun C (2023). Loss of cervical sagittal alignment worsens the cervical spinal lesions in patients with Hirayama Disease. Neurol Sci.

[5] Zheng C, Nie C, Lei W (2018). CAN anterior cervical fusion procedures prevent the progression of the natural course of Hirayama Disease? An ambispective cohort analysis. Clin Neurophysiol.

[6] Xu X, Han H, Gao H (2011). The increased range of cervical flexed motion detected by radiographs in Hirayama Disease. Eur J Radiol.

[7] Lu X, Zou F, Lu F (2022). How to reconstruct the lordosis of cervical spine in patients with Hirayama Disease? A finite element analysis of biomechanical changes focusing on adjacent segments after anterior cervical discectomy and fusion. J Orthop Surg Res.

[8] Park MS, Kelly MP, Lee DH (2014). Sagittal alignment as a predictor of clinical adjacent segment pathology requiring Surgery after anterior cervical arthrodesis. Spine J.

[9] Kong L, Sun C, Kou N (2018). Risk factors associated with clinical adjacent segment pathology following multi-level cervical fusion Surgery. Med (Baltim).

[10] Alpayci M, Şenköy E, Delen V (2016). Decreased neck muscle strength in patients with the loss of cervical lordosis. Clin Biomech (Bristol Avon).

[11] Alpayci M, İlter S (2017). Isometric Exercise for the cervical extensors can help restore physiological lordosis and reduce Neck Pain: a Randomized Controlled Trial. Am J Phys Med Rehabil.

[12] Tian Y, Xie L, Jiang J (2022). Why the patients with Hirayama Disease have abnormal cervical sagittal alignment? A radiological measurement analysis of posterior cervical extensors. J Orthop Surg Res.

[13] Ainsworth BE, Haskell WL, Whitt MC (2000). Compendium of physical activities: an update of activity codes and MET intensities. Med Sci Sports Exerc.

[14] Caspersen CJ, Powell KE, Christenson GM (1985). Physical activity, exercise, and physical fitness: definitions and distinctions for health-related research. Public Health Rep.

[15] Nie C, Chen K, Zhu YU (2022). Comparison of time-dependent resistance isometric exercise and active range of motion exercise in alleviating the sensitization of postoperative axial pain after cervical laminoplasty. Musculoskelet Sci Pract.

[16] Zheng C, Zhu Y, Zhu D (2017). Motor unit number estimation in the quantitative assessment of severity and progression of motor unit loss in Hirayama Disease. Clin Neurophysiol.

[17] Bromberg MB, Brownell AA (2008). Motor unit number estimation in the assessment of performance and function in motor neuron Disease. Phys Med Rehabil Clin N Am.

[18] Fukada K, Matsui T, Furuta M (2016). The Motor Unit Number Index of subclinical abnormality in Amyotrophic Lateral Sclerosis. J Clin Neurophysiol.

[19] Gawel M, Kostera-Pruszczyk A (2014). Effect of age and gender on the number of motor units in healthy subjects estimated by the multipoint incremental MUNE method. J Clin Neurophysiol.

[20] Toma S, Shiozawa Z (1995). Amyotrophic cervical myelopathy in adolescence. J Neurol Neurosurg Psychiatry.

[21] Kohno M, Takahashi H, Yagishita A (1998). Disproportion theory of the cervical spine and spinal cord in patients with juvenile cervical flexion myelopathy. A study comparing cervical magnetic resonance images with those of normal controls. Surg Neurol.

[22] Chen CJ, Chen CM, Wu CL (1998). Hirayama Disease: MR Diagnosis. AJNR Am J Neuroradiol.

