# Supplementary material for: The role of leisure-time physical activity in maintaining cervical lordosis after anterior cervical fusion and its impact on the motor function in patients with hirayama disease: a retrospective cohort analysis
Source: BMC Musculoskelet Disord. 2023 Nov 21;24:903. doi: 10.1186/s12891-023-07038-w (PMC10662470; doi:10.1186/s12891-023-07038-w)
Supplement: Supplementary file 1 — Supplementary Material 1: Supplementary Table 1 [file 12891_2023_7038_MOESM1_ESM.pdf]

Supplementary Table 1: MET values and formula for measuring IPAQ scores

	MET values and formula
**Work Domain**	
Walking MET·min/w	3.3 × walking minutes × walking days at work
Moderate MET·min/w	4.0 × moderate-intensity activity minutes × moderate-intensity days at work
Vigorous MET·min/w	8.0 × vigorous-intensity activity minutes × vigorous-intensity days at work
Total Work	Walking + Moderate + Vigorous MET·min/w at work
**Active Transportation Domain**	
Walking MET·min/w	3.3 × walking minutes × walking days for transportation
Cycle MET·min/w	6.0 × cycling minutes × cycle days for transportation
Total Transport	Walking + Cycling MET·min/w for transportation
**Domestic and Garden Domain**	
Vigorous MET·min/w	5.5 × vigorous-intensity activity minutes × vigorous-intensity days do garden work
Moderate MET·min/w	4.0 × moderate-intensity activity minutes × moderate-intensity days do garden work
Moderate MET·min/w	3.0 × moderate-intensity activity minutes × moderate-intensity days do inside chores
Total Domestic and Garden	Vigorous garden + Moderate garden + Moderate inside chores MET·min/w
**Leisure-Time Domain**	
Walking MET·min/w	3.3 × walking minutes × walking days in leisure
Moderate MET·min/w	4.0 × moderate-intensity activity minutes × moderate-intensity days in leisure
Vigorous MET·min/w	8.0 × vigorous-intensity activity minutes × vigorous-intensity days in leisure
Total Leisure-Time	Walking + Moderate + Vigorous MET·min/w in leisure
**Total Physical Activity Scores**	
Total physical activity	Total Work + Total Transport + Total Domestic and Garden + Total Leisure-Time

IPAQ: International Physical Activity Questionnaire
