# Supplementary material for: The role of leisure-time physical activity in maintaining cervical lordosis after anterior cervical fusion and its impact on the motor function in patients with hirayama disease: a retrospective cohort analysis
Source: BMC Musculoskelet Disord. 2023 Nov 21;24:903. doi: 10.1186/s12891-023-07038-w (PMC10662470; doi:10.1186/s12891-023-07038-w)
Supplement: Supplementary file 2 — Supplementary Material 2: Supplementary Table 2 [file 12891_2023_7038_MOESM2_ESM.pdf]

Supplementary Table 2：Preoperative measurements between the HD patients with and without neck-standard LOA

	**Patients with neck-standard LOA**	**Patients without neck-standard LOA**
**Number of patients**	15	76
**MUNE assessments**		
Symptomatic CMAP (mV)	5.0 ± 2.4	6.5 ± 2.8
Symptomatic SMUP (μV)	163.7 ± 95.6*	100.0 ± 50.3*
Symptomatic motor units	43.4 ± 38.3*	85.2 ± 58.7*
Less-symptomatic CMAP (mV)	7.0 ± 2.6*	8.7 ± 2.8*
Less-symptomatic SMUP (μV)	115.6 ± 44.9*	80.7 ± 42.3*
Less-symptomatic motor units	75.9 ± 60.3*	141.3 ± 84.4*
**Clinical functional assessments**		
Symptomatic HGS (Kg)	21.9 ± 10.8	24.8 ± 11.0
Less-symptomatic HGS (Kg)	29.7 ± 9.4	32.9 ± 8.9
DASH	9.1 ± 8.8	8.4 ± 6.7
**Imaging assessments**		
C2-7 Cobb (degrees)	-5.7 ± 9.6*	8.5 ± 10.6*
CSA#	8.2 ± 1.4 (8/55)	9.1 ± 1.9 (47/55)
FI#	0.27 ± 0.08 (8/55)	0.22 ± 0.08 (47/55)

Measurements are expressed as the mean ± SD

*: Statistically significant differences between the HD patients with and without neck-standard LOA

a/b: Number of patients with or without neck-standard LOA/number of total patients accepting imaging assessments

HD: Hirayama disease; **LOA:** Loss of dorsal dural attachment from the pedicle; **CSA:** Cross-sectional area of posterior cervical muscles; **FI:** Fatty infiltration of posterior cervical muscles; **MUNE:** Motor unit number estimation; **CMAP:** Compound muscle action potential; **SMUP:** Single motor unit potential; **HGS:** Handgrip strength; **DASH:** The disabilities of the arm, shoulder and hand outcome measure;
