# Supplementary material for: The role of leisure-time physical activity in maintaining cervical lordosis after anterior cervical fusion and its impact on the motor function in patients with hirayama disease: a retrospective cohort analysis
Source: BMC Musculoskelet Disord. 2023 Nov 21;24:903. doi: 10.1186/s12891-023-07038-w (PMC10662470; doi:10.1186/s12891-023-07038-w)
Supplement: Supplementary file 3 — Supplementary Material 3: Supplementary Table 3 [file 12891_2023_7038_MOESM3_ESM.pdf]

Supplementary Table 3: The main types of exercises of each HD patients performing LTPA

The main types	Number of patients (n/total patient (%))
Walking	3/28 (10.7%)
Brisk walking	2/28 (7.1%)
Jogging	5/28 (17.9%)
Climbing (by stairs)	1/28 (3.6%)
Playing football	1/28 (3.6%)
Cycling	3/28 10.7%)
Swimming	1/28 (3.1%)
Calisthenics (e.g., HIIT)	3/28 (10.7%)
Flying a kite	1/28 (3.6%)
Playing basketball	1/28 (3.6%)
Playing badminton	2/28 (7.1%)
Strength training	4/28 (14.3%)
Wushu	1/28 (3.6%)

HD: Hirayama disease; **LTPA:** Leisure-time physical activities; **HIIT:** High intensity intermittent aerobic
