# Supplementary material for: The role of leisure-time physical activity in maintaining cervical lordosis after anterior cervical fusion and its impact on the motor function in patients with hirayama disease: a retrospective cohort analysis
Source: BMC Musculoskelet Disord. 2023 Nov 21;24:903. doi: 10.1186/s12891-023-07038-w (PMC10662470; doi:10.1186/s12891-023-07038-w)
Supplement: Supplementary file 4 — Supplementary Material 4: Supplementary Table 4 [file 12891_2023_7038_MOESM4_ESM.pdf]

Supplementary Table 4: Measurements between the HD patients with and without LTPA

	**Patients with LTPA**	**Patients without LTPA**
**Number of patients**	28	63
**Preoperative motor functional assessments**		
Symptomatic CMAP (mV)	5.7 ± 2.6	6.5 ± 2.8
Symptomatic SMUP (μV)	109.4 ± 64.4	111.0 ± 64.3
Symptomatic motor units	66.3 ± 48.0	83.7 ± 61.3
Less-symptomatic CMAP (mV)	8.1 ± 2.7	8.6 ± 2.9
Less-symptomatic SMUP (μV)	86.8 ± 51.1	86.3 ± 41.6
Less-symptomatic motor units	128.6 ± 82.7	131.4 ± 85.6
Symptomatic HGS (Kg)	21.3 ± 8.8	25.7 ± 11.7
Less-symptomatic HGS (Kg)	29.7 ± 8.3	33.6 ± 9.1
DASH	9.8 ± 7.2	7.9 ± 6.9
**Preoperative imaging assessments**		
C2-7 Cobb before operation	9.1 ± 12.4	4.9 ± 11.2
CSA#	8.3 ± 1.3 (14/55)	9.2 ± 2.0 (41/55)
FI#	0.26 ± 0.09 (14/55)	0.22 ± 0.08 (41/55)
**Postoperative motor functional assessments**		
Symptomatic CMAP (mV)	6.2 ± 2.6	6.5 ± 2.8
Symptomatic SMUP (μV)	116.2 ± 92.4	107.6 ± 68.3
Symptomatic motor units	76.0 ± 57.2	84.0 ± 58.3
Less-symptomatic CMAP (mV)	8.4 ± 3.0	8.6 ± 2.9
Less-symptomatic SMUP (μV)	76.8 ± 37.1	89.4 ± 45.8
Less-symptomatic motor units	136.8 ± 78.2	133.0 ± 89.6
Symptomatic HGS (Kg)	23.1 ± 8.6	25.5 ± 10.7
Less-symptomatic HGS (Kg)	29.7 ± 8.2	33.5 ± 8.9
DASH	8.9 ± 6.8	7.7 ± 6.8
**Postoperative imaging assessments**		
C2-7 Cobb immediate after operation	13.7 ± 11.1	11.4 ± 9.8
C2-7 Cobb 2 years after operation	16.8 ± 11.4*	9.4 ± 9.4*
CSA#	8.2 ± 2.1 (14/55)	8.9 ± 3.0 (41/55)
FI#	0.24 ± 0.07 (14/55)	0.24 ± 0.05 (41/55)

Measurements are expressed as the mean ± SD

#: These measurements of the imaging assessments were recorded from 55 of 91 HD patients.

*: Statistically significant differences between the patients with and without LTPA

a/b: Number of patients with or without LTPA/number of total patients accepting imaging assessments

HD: Hirayama disease; **LTPA:** Leisure-time physical activities; **CSA:** Cross-sectional area of posterior cervical muscles; **FI:** Fatty infiltration of posterior cervical muscles; **CMAP:** Compound muscle action potential; **SMUP:** Single motor unit potential; **HGS:** Handgrip strength; **DASH:** The disabilities of the arm, shoulder and hand outcome measure
