# Supplementary material for: The role of leisure-time physical activity in maintaining cervical lordosis after anterior cervical fusion and its impact on the motor function in patients with hirayama disease: a retrospective cohort analysis
Source: BMC Musculoskelet Disord. 2023 Nov 21;24:903. doi: 10.1186/s12891-023-07038-w (PMC10662470; doi:10.1186/s12891-023-07038-w)
Supplement: Supplementary file 5 — Supplementary Material 5: Supplementary Table 5 [file 12891_2023_7038_MOESM5_ESM.pdf]

Supplementary Table 5: The relationship (P-values) between Leisure-Time IPAQ values and both the postoperative measurements and the postoperative changes of these measurements in HD patients

	IPAQ values of LTPA
**Postoperative imaging assessments**	
Postoperative 2-year C2-7 Cobb	0.99
CSA	0.13
FI	0.78
**Postoperative changes of imaging assessments**	
C2-7 Cobb before and 2 years after operation	0.34
C2-7 Cobb immediate and 2 years after operation	0.19
CSA	0.18
FI	1.00
**Postoperative motor functional assessments**	
Symptomatic CMAP (mV)	0.89
Symptomatic SMUP (μV)	0.30
Symptomatic motor units	0.84
Less-symptomatic CMAP (mV)	0.84
Less-symptomatic SMUP (μV)	0.14
Less-symptomatic motor units	0.44
Symptomatic HGS (Kg)	0.98
Less-symptomatic HGS (Kg)	0.93
DASH	0.29
**Postoperative changes of imaging assessments**	
Symptomatic CMAP (mV)	0.15
Symptomatic SMUP (μV)	0.42
Symptomatic motor units	0.08
Less-symptomatic CMAP (mV)	0.17
Less-symptomatic SMUP (μV)	0.92
Less-symptomatic motor units	0.07
Symptomatic HGS (Kg)	0.62
Less-symptomatic HGS (Kg)	0.26
DASH	0.60

HD: Hirayama disease; **LTPA:** Leisure-time physical activities; **CSA:** Cross-sectional area; **FI:** Fatty infiltration of posterior cervical muscles; **CMAP:** Compound muscle action potential; **SMUP:** Single motor unit potential; **HGS:** Handgrip strength; **DASH:** The disabilities of the arm, shoulder and hand outcome measure; **IPAQ:** International Physical Activity Questionnaire
