# Supplementary figures and images for: The role of leisure-time physical activity in maintaining cervical lordosis after anterior cervical fusion and its impact on the motor function in patients with hirayama disease: a retrospective cohort analysis
Source: BMC Musculoskelet Disord. 2023 Nov 21;24:903. doi: 10.1186/s12891-023-07038-w (PMC10662470; doi:10.1186/s12891-023-07038-w)

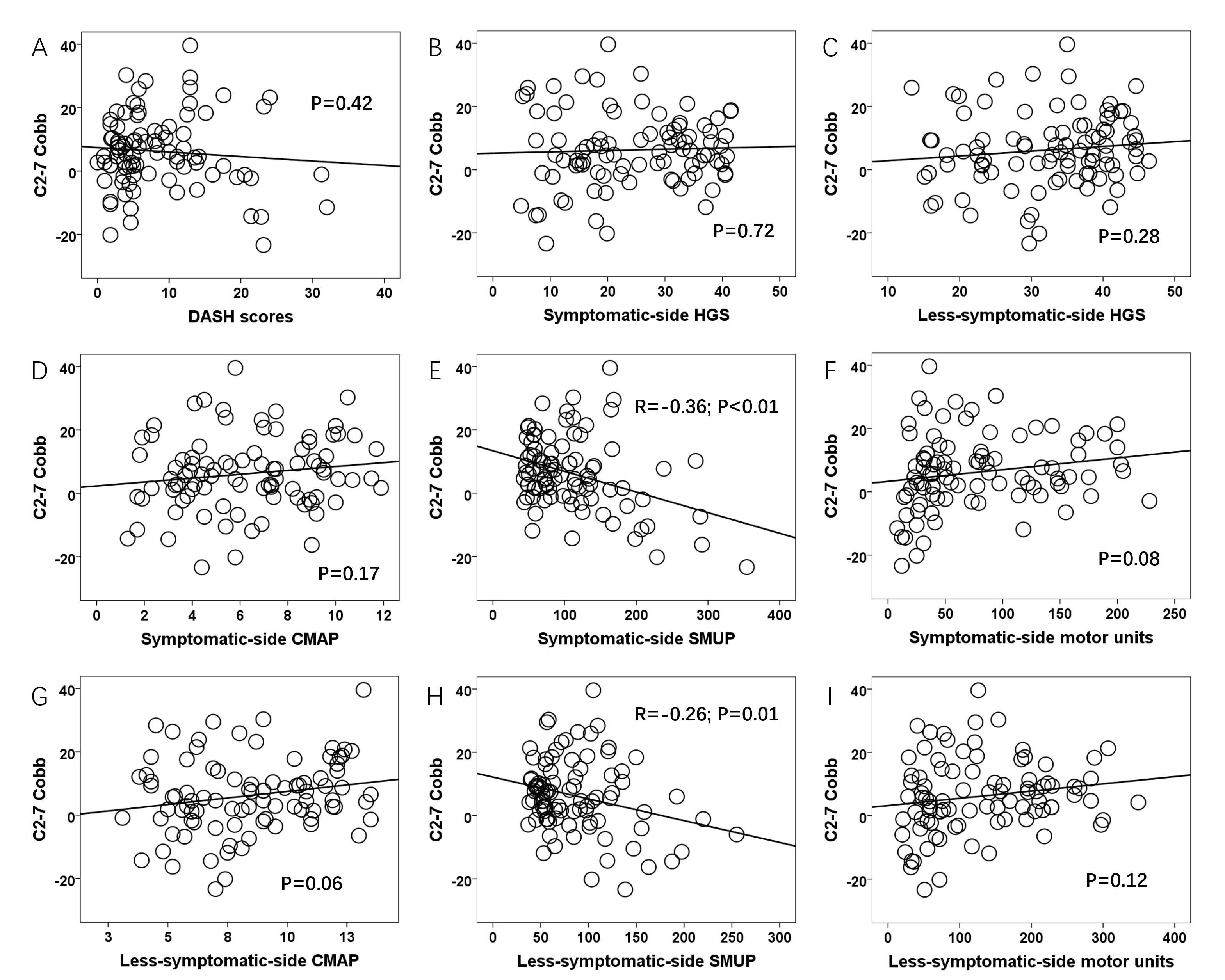

Supplement: Supplementary file 6 — Supplementary Material 6: Supplementary Figure 1 [file 12891_2023_7038_MOESM6_ESM.jpg]

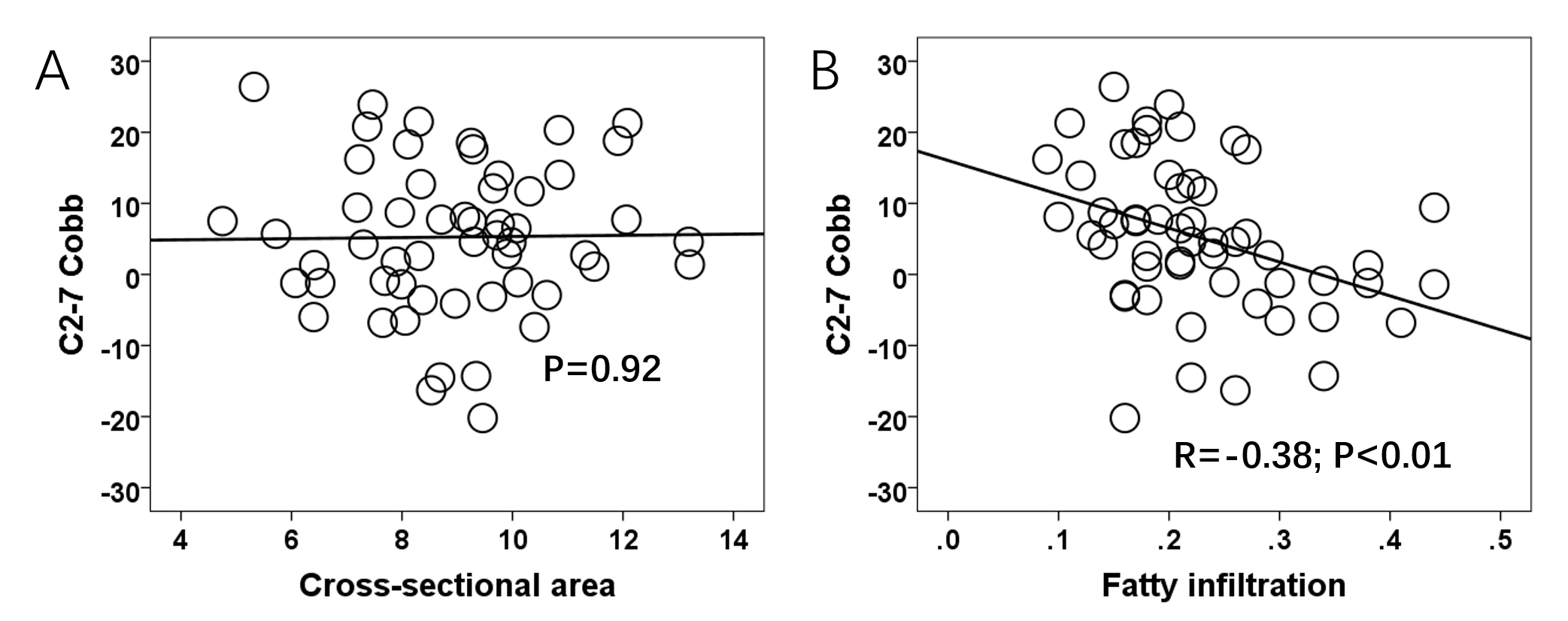

Supplement: Supplementary file 7 — Supplementary Material 7: Supplementary Figure 2 [file 12891_2023_7038_MOESM7_ESM.jpg]
