# Supplementary material for: The role of leisure-time physical activity in maintaining cervical lordosis after anterior cervical fusion and its impact on the motor function in patients with hirayama disease: a retrospective cohort analysis
Source: BMC Musculoskelet Disord. 2023 Nov 21;24:903. doi: 10.1186/s12891-023-07038-w (PMC10662470; doi:10.1186/s12891-023-07038-w)
Supplement: Supplementary file 9 — Supplementary Material 9: Supplementary figures legend [file 12891_2023_7038_MOESM9_ESM.docx]

**Figure Legends:**

**Supplementary Fig. 1:** Correlation between preoperative C2-7 Cobb and preoperative measurements of the motor function assessments in HD patients. There was a negative correlation between the preoperative C2-7 Cobb and bilateral average SMUP amplitudes (**E, H**). There was no correlation between the C2-7 Cobb and DASH scores (**A**), bilateral HGS (**B, C**), bilateral CMAP amplitudes (**D, G**) and bilateral number of motor units (**F, I**). **HD:** Hirayama disease; **CMAP:** Compound muscle action potential; **SMUP:** Single motor unit potential; HGS: Handgrip strength; **DASH:** The disabilities of the arm, shoulder and hand outcome measure; **P:** P-values.

**Supplementary Fig. 2:** Correlation between preoperative C2-7 Cobb and preoperative cross-sectional area and fatty infiltration of the posterior cervical muscles in HD patients. The C2-7 Cobb was negatively associated with fatty infiltration of the posterior cervical muscles (**B**). There was no correlation between the C2-7 Cobb and cross-sectional area of the posterior cervical muscles (**A**). **HD:** Hirayama disease; **P:** P-values.
